# Why did the UV-A-induced photoluminescent blue–green glow in trilobite eyes and exoskeletons not cause problems for trilobites?

**DOI:** 10.7717/peerj.1492

**Published:** 2015-12-15

**Authors:** Brigitte Schoenemann, Euan N.K. Clarkson, Gábor Horváth

**Affiliations:** 1 Department of Animal Physiology and Institute of Biology Education (Zoology),University of Cologne, Cologne, Germany; 2 Grant Institute, University of Edinburgh, Edinburgh, United Kingdom; 3 Environmental Optics Laboratory, Department of Biological Physics, Physical Institute,Eötvös Loránd University, Budapest, Hungary

**Keywords:** Vision, Trilobite, Arthropod, UV-radiation, Luminescene, Optics, Compound eye, Cambrian explosion, Palaeozoic, Calcite

## Abstract

The calcitic lenses in the eyes of Palaeozoic trilobites are unique in the animal kingdom, although the use of calcite would have conveyed great advantages for vision in aquatic systems. Calcite lenses are transparent, and due to their high refractive index they would facilitate the focusing of light. In some respects, however, calcite lenses bear evident disadvantages. Birefringence would cause double images at different depths, but this is not a problem for trilobites since the difference in the paths of the ordinary and extraordinary rays is less than the diameter of the receptor cells. Another point, not discussed hitherto, is that calcite fluoresces when illuminated with UV-A. Here we show experimentally that calcite lenses fluoresce, and we discuss why fluorescence does not diminish the optical quality of these lenses and the image formed by them. In the environments in which the trilobites lived, UV-A would not have been a relevant factor, and thus fluorescence would not have disturbed or confused their visual system. We also argue that whatever the reason that calcite was never again used successfully in the visual systems of aquatic arthropods, it was not fluorescence.

## Introduction

Trilobites were the most prevalent mobile invertebrates of the Palaeozoic seas, as is known from their fossilised remains. They were arthropods, equipped with a thick shell and highly differentiated compound eyes from the very beginning of their appearance in the fossil record, some 522 million years ago. Trilobites developed a very special optical system, contrasting with those of all other arthropods. Uniquely in the animal realm, they had compound eyes with lenses of oriented calcite rather than of organic material (Towe, 1973; Clarkson, Levi-Setti & Horváth, 2006; Lee, Torney & Owen, 2007; Lee, Torney & Owen, 2012). The use of calcite brings an evident advantage optically, especially for aquatic organisms. The high refractive index of calcite (∼590 nm: *n*_*ω*_ = 1.640–1.660, *n*_ε_ = 1.486) by contrast with that of chitin, the lens material of most other arthropods (*n* = 1.46, rarely up to 1.56 (Land & Nilsson, 2012)), increases the difference in refractive indices between the visual system of the arthropod and water (*n* = 1.334, seawater), and thus facilitates focusing due to strong refraction. A point of special interest has been the visual system of a suborder of trilobites, the Phacopina, because their large lenses (diameters of up to 2 mm and more in e.g., *Drotops megalomanicus* Struve, 1990) have an elegant internal substructure which probably corrected lens aberrations (especially spherical aberration) which would otherwise be produced by the thick lenses that phacopid trilobites possess (Clarkson & Levi-Setti, 1975). Although nothing is usually preserved below the level of the lenses, the first known sublensar sensory structures at a cellular level have been very recently described in these trilobites (Schoenemann & Clarkson, 2013). This raises questions about the specificity of this unique calcitic system, which persisted successfully for more than 250 million years but was never reinvented again after trilobites became extinct, despite the high advantage of transparency and a high refractive index which allows efficient focusing even under water.

Calcite is a strongly birefringent mineral, and light passing through it in directions other than parallel with the *c*-axis splits into two rays, producing double images at different depths. At first sight this may seem to be a problem for trilobite vision. However, because the difference of paths in the ordinary and extraordinary ray on their way through the lens is smaller than the separation of common photoreceptor units (being usually larger than the receptor diameter), the double images may be irrelevant (Schoenemann & Clarkson, 2011).

Another striking characteristic of the mineral calcite, apart from birefringence, is photoluminescence. The photoluminescence is usually related to impurities of organic material or minerals, such as magnesium, manganese, iron etc. as well as cracks (Machel, 1985; Machel et al., 1991; Pedone, Cercone & Burruss, 1990). Natural calcite fluoresces when it is illuminated with light of certain wavelengths, as for example UV-light, and the colour of this fluorescence depends on the character of the particles the calcite includes. The energy of the incident light is able to excite susceptible electrons within the atomic structure of the mineral. They leave their position and jump to higher orbits of the atomic structure. Falling back, they release a small amount of energy visible as light, and produce a kind of ‘glow.’ The colour of this ‘glow’ is often different from the colour of the incident light, and depends on the composition of the calcite, while the ‘glow’ continues as long as the mineral is illuminated. The colour of the glow depends on the orbit from which the electron returns to its original position. In contrast, during phosphorescence the light is ‘stored’ for a while inside the atomic structure; the system becomes ‘charged,’ and releases the energy more slowly than during the fluorescent process. The excited electron also returns to its position inside the atomic structure but undergoes certain intersystem levels, while its state of spin turns to a higher spin multiplicity, normally a triplet state. These transitions take time in the order of milliseconds, but can also persist in some materials for minutes or even hours. In our probe the phosphorescence, seen in a biological time scale (milliseconds), disappears as soon as the light vanishes. While calcite shares this property of showing fluorescence with numerous other natural minerals such as fluorites or opals as well as synthetic minerals (Nakamura et al., 2013), at a first glance it seems quite extraordinary to find a presumably fluorescent mineral element in the morphology of a biological system, especially a visual system.

Calcium carbonate exists in many biological systems. For example, in the form of calcite it is reported from light-sensitive systems in brittle stars (Aizenberg et al., 2001), the shells of brachiopods, ostracodes and other crustaceans (Xia, Ito & Engstrom, 1997). On the other hand, the shells of many kinds of molluscs are built of aragonite, a form of calcium carbonate with a crystal lattice different from that of calcite, and typical for the exoskeletons of corals and some serpulids. Calcium carbonate (calcite) is not known so far in image-forming structures, except in trilobites.

Bioluminescence occurs widely in living systems, especially in marine vertebrates, invertebrates, some fungi, and many microorganisms, but not in land vertebrates and higher plants. There is a distinction between primary bioluminescence, where the organism itself generates the light, and secondary bioluminescence, where the light is produced by symbiotic microorganisms which are themselves primary bioluminescants. A very common, basic system is the oxidation of luciferin by the enzyme luciferase; there are other enzymes involved such as superphotoxidase in fungi (Shimomura, 1992, Desjardin, Oliveira & Stevani, 2008), or aequorin in the jellyfish *Aequorea victoria* (Hastings, 1983; Kendall & Badminton, 1998, Shimomura, 2005, Gruber & Pieribone, 2007; Meyer-Rochow, 2009; Haddock, Moline & Case, 2010; Sparks et al., 2014). Bioluminescence is used to attract mating partners, for defence, warning, mimicry, and for illumination or as counterillumination balancing the residual downwelling light to cloak the silhouette from upward-looking predators, as was recently reported for bioluminescent sharks (Claes et al., 2014). Whether bioluminescence is useful, especially fluorescence in a visual organ, such as is caused by UV-light in the calcitic lenses of the dioptric apparatus in trilobite compound eyes, may be worth further consideration.

The precise analysis of different trilobite lenses has shown that during diagenesis the composition of the calcitic lenses of different trilobites has been altered (Lee, Torney & Owen, 2007; Lee, Torney & Owen, 2012); this becomes very evident in the meanwhile famous red trilobites with green eyes from Morocco, which had undergone a silicified preservation rather than a fossilisation in limestone as is more or less usual (Klug, Schulz & De Baets, 2009). The Hunsrück Slate is well known for its exceptional preservation and that calcium carbonate is often dissolved or replaced. As is shown here, however, we still see fluorescence of the lenses even today, so it seems allowed to assume that there exists no pervasive diagenetic influence on this system. It will not be possible, however, to reconstruct the precise original mineral composition of the lenses. Consequently, the actual character of the fluorescence in the lenses of trilobite compound eyes during the life-times of the trilobites remains unknown, but some discussion of the relevance of the potential phenomenon of fluorescence in these ancient calcitic lenses, in principle, would seem desirable. In fact, there are three main questions which it seems worthwhile to answer:

1. Do the lenses of trilobite eyes, after all this theoretical discussion, really show fluorescence?
2. What are the optical and sensory consequences of fluorescence, if this is indeed what they actually show?
3. Is the reason why calcite has not been used more often in aquatic optical systems is the fact that it is fluorescent?

## Materials and Methods

**Figure 1:**
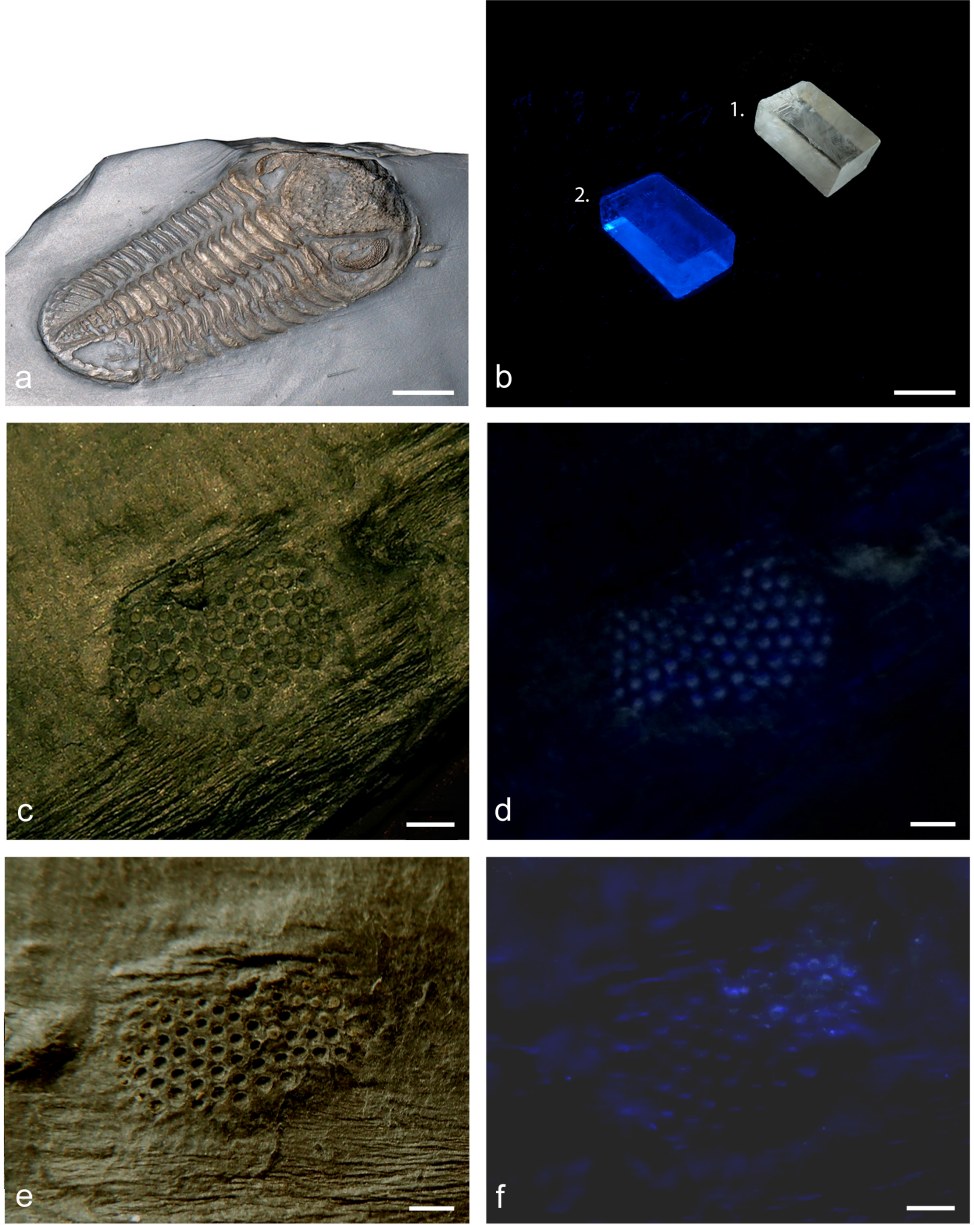
The glow in the calcitic lenses of a phacopid trilobite’s eye. (A) *Chotecops ferdinandi* (Kayser, 1880), Bundenbachschiefer, Lower Devonian, Location: Grube Eschenbach (?), Hunsrück, Germany, scale bar ∼ 1 cm. (Coll. Boettcher 2014, housed in the collection of Steinmann Istitute, University of Bonn (STIPB-AR-075) (B) 1, Calcite crystal (∼3 cm); 2, Fluorescent when illuminated with ∼365 nm under water. (C) Isolated moult of a *Chotecops* compound eye with lenses preserved (GIK 2118). (D) The same showing fluorescence in the calcitic lenses of the trilobite compound eye when illuminated with UVA-light (∼365 nm). (E) Isolated moult of a *Chotecops* compound eye with lenses preserved (GIK 2119). (D) The same showing fluorescence in the calcitic lenses of the trilobite compound eye when illuminated with UVA-light (365 nm). (B–F) Scale bar ∼ 1 mm.

Because most trilobite exoskeletons fossilised in limestones are largely composed of calcite, experiments for investigating the photoluminescence of calcite lenses were performed on a species which normally fossilises in a somewhat different way. The specimens used here come from the Bundenbachschiefer of the Lower Devonian of the Hunsrück region, Germany. In these trilobites, the sulfur released from proteins together with iron from the ancient mud formed pyrite, while the lenses of pure calcite stayed as they were. The phacopid trilobite *Chotecops ferdinandi* (Kayser, 1880) (Fig. 1A) is very abundant at this location and possesses large (∼7 mm) compound eyes. Lens preservation, however, is extremely rare because the lenses normally fall out of the fossil, and cavities remain where the lenses had been. Even so, very occasional examples are found such as the two isolated eyes of moulted specimens used here, each showing the phenomenon independently(Figs. 1C–1F). Detailed reports about the age and setting of the Hunsrück Slate fauna, taphonomy and lithostratigraphy are given in e.g., Schindler et al. (2002), Kühl et al. (2012) and De Baets et al. (2013). The specimens are housed in the collection of the Geological Institute of the University of Cologne (now Institute of Geology and Mineralogy). The museum numbers are GIK 2118 and GIK 2119. They were illuminated with a peak-wavelength of ∼365 nm (UV-A: specification of the manufacturer) from a source of low energy (6 V, 4 W, 40 mA, ETT Comp. Braunschweig, Germany, specification of the manufacturer) and photographed (Panasonic DMC-TZ10). The width of the spectrum of the light source is unknown and is not relevant for showing the principal phenomenon of fluorescence in the calcitic lenses of phacopid trilobite compound eyes.

## Results

When illuminated with UV-A light (365 nm) the remains of the calcitic lenses glow with a blue-greenish light as long as they are illuminated, while other parts of the eye, which are not of lens material, remain (more or less) dark. Both of the extremely rare specimens show the phenomenon in the same way and independently.

## Discussion

The fact that the material of trilobite lenses was primary calcite, as proposed by Towe (1973), has been unequivocally confirmed; the lenses of all known species were originally calcitic, independently of how they have been preserved (Clarkson, 1975; Clarkson, 1979; Clarkson, 1997; Clarkson, Levi-Setti & Horváth, 2006). This understanding has been strengthened by the use of mineralogical methods and particularly by the use of Electron Backscattered Diffraction (EBSD) technology (Lee, Torney & Owen, 2007; Lee, Torney & Owen, 2012). These Lower Devonian compound eyes investigated here are almost 400 million years old. As already mentioned, the mineral content may have changed during preservation and possible recrystallisation, and consequently the colour of fluorescence and its intensity may have changed. Whereas it will probably never be possible to reconstruct the original composition precisely, the potential to generate the phenomenon itself in principle, however, is clearly shown in Fig. 1, where the calcitic lenses so evidently fluoresce. So the first and basic question, whether there is really some potential in the calcitic lenses of trilobite eyes to produce fluorescence, can be answered positively.

What are the optical and sensory consequences of such fluorescence?

It seems necessary to first consider what would be the consequences for the visual system, if we had a pure perception of UV-A light and no other.

**Figure 2:**
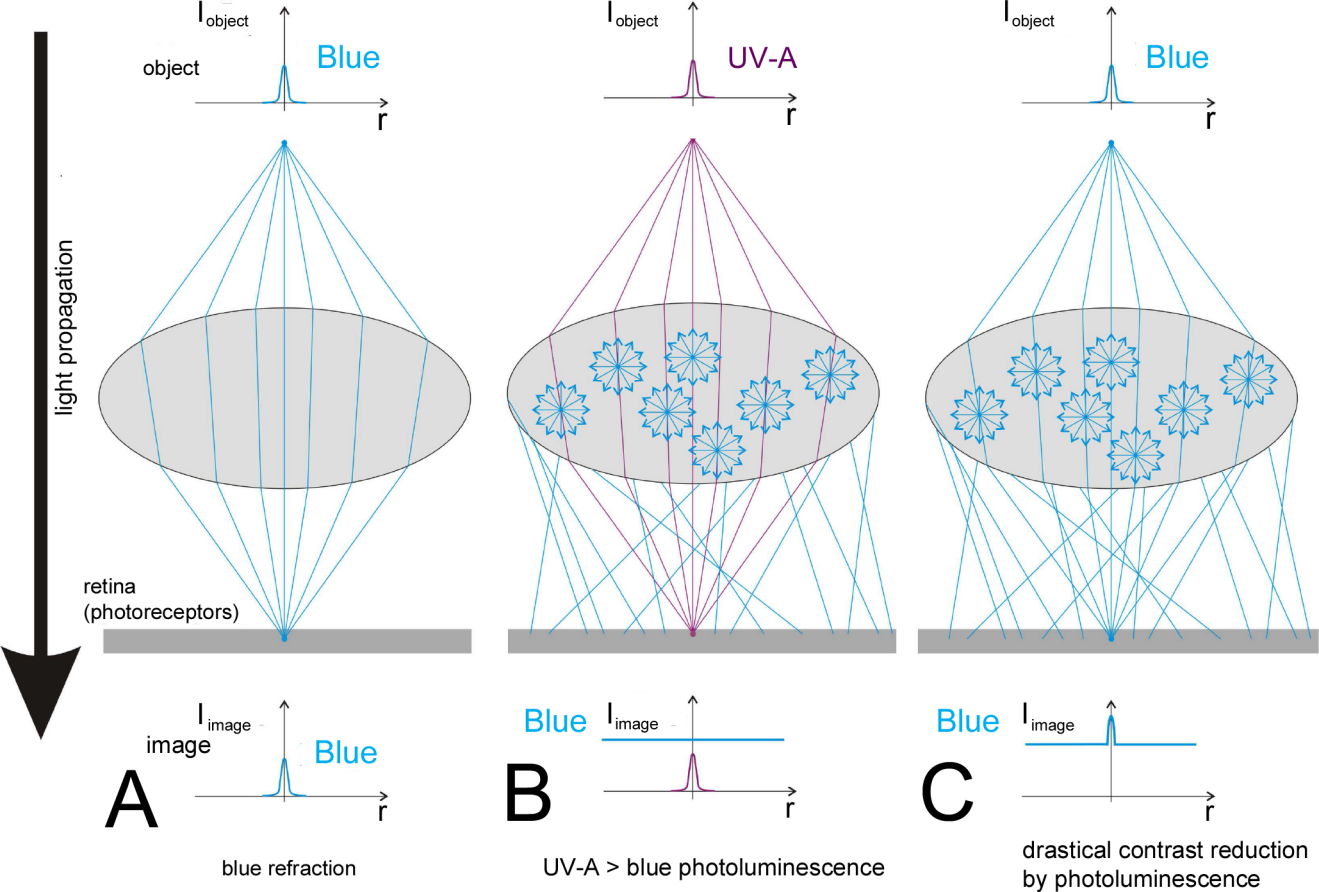
The optical problem caused by the UV-A-induced photoluminescent diffuse blue light in the image formation by a dioptric apparatus. For further explanations, see text.

As Fig. 2A shows, the normal function of a lens is to focus incident light to one point. We do not know exactly what the underlying sensory system in a trilobite’s compound eye actually was. It is rather probable that under each lens of the compound eye, which from outside is recognisable as a facet, was a so-called ommatidium, as we find it in apposition compound eyes of many diurnal arthropods living today such as dragonflies or bees. It is the oldest system of compound eyes; more advanced systems adapted to dimmer light conditions probably did not evolve before the Devonian (Gaten, 1998). In the apposition eyes the light is focused through a normally chitinous lens, or structure functioning as such, onto a central light guiding structure, the so-called rhabdom, which is part of several (often eight) photoreceptor cells. In the rhabdom lie the photopigments, and the energy of the incident light alters the sterical form of the photopigments to evoke an electrical signal which can be processed by the nervous system of the organism. The ommatidia are isolated from each other by pigment cells. Because the rhabdom integrates all optical inputs inside the angle of view of the ommatidium, there results over the entire compound eye a mosaic-like image. The higher the number of facets, the more acute is the image, in the same way that pixels contribute to a computer graphic, and the smaller the field of view of each ommatidium. An indication that trilobites had a kind of apposition compound eye was described recently using X-ray tomography and synchrotron radiation in phacopid trilobites (Schoenemann & Clarkson, 2013). An alternative to this system is the establishment of a small retina, a layer of receptors below the lens, as we know it among arthropods from myriapods and many chelicerates. If there was a third alternative, it would not yet be known.

In principle, this mosaic-like character of the image formed by an apposition eye should more or less be retained by any fluorescent pattern of the compound eyes’ lenses generated by an inhomogenous UV-light distribution in the environment, but because in sum all points of fluorescence inside the lens cause a high loss of contrast, this principle cannot be entirely adopted, as we shall see.

Figure 2 shows what happens when UV-A light enters the calcitic lenses of a trilobite. An object point is characterized by the intensity function *I*_object_(*r*), where *r* is the radius measured perpendicularly from the optical axis. It should be projected onto the light-sensitive receptor plane as a sharp image point, the function *I*_image_(*r*) of which is similar to *I*_object_(*r*). Sharpness means that the narrow and high intensity peak of *I*_object_(*r*) is transferred by the dioptric apparatus as a similarly narrow and similarly high intensity peak of *I*_image_(*r*) as seen in Fig. 2A for blue light, characteristic of the semi-monochromatic optical environment of trilobites.

A point source of UV-A light is similarly imaged onto the retina as shown in Fig. 2B. But UV-A induces blue(-green) light in the bulk calcite medium of the lens. This UV-A-induced blue light propagates in all possible directions from its numerous point sources in the lens. After refraction on the lens surface, this diffuse blue light reaches the sensory system below, where it forms a relatively intense, practically homogeneous blue background light field *I*_blue_(*r*) = constant (Fig. 2B). Thus, the sharp-peaked object point with *I*_object_(*r*) is projected as a wide blue circular spot with a small intensity peak in its center, as shown in Fig. 2C.

This would happen to each of the tesserae in the mosaic-like vision of a trilobite compound eye with an assumed apposition eye system. It would destroy the integrating properties of the rhabdom because of a loss of intensity in its received signal. Over the whole compound eye this would result in a loss of contrast.

If we had a retinal system below the lens, this mechanism would help to supply all receptor cells of this visual unit with light—but then the question rises as to why there is a sometimes probably even sophisticated lens with a central focusing (Clarkson & Levi-Setti, 1975). A light distribution as results to *I*_image_(*r*) would be of help just in a combined system, something with a centralised visual system like a fovea/or ommatidium and peripherally a supportive system with retinal receptor cells.

Another disadvantage might be that the UV-A light from nearly all angles would enter the lens, and thus the fluorescence would be roughly equal in all lenses of the eye, irrespective of which part of the visual field the light came from. Any image formation would be corrupted, and the details of the environment would be no more resolved than just light or no light.

**Figure 3:**
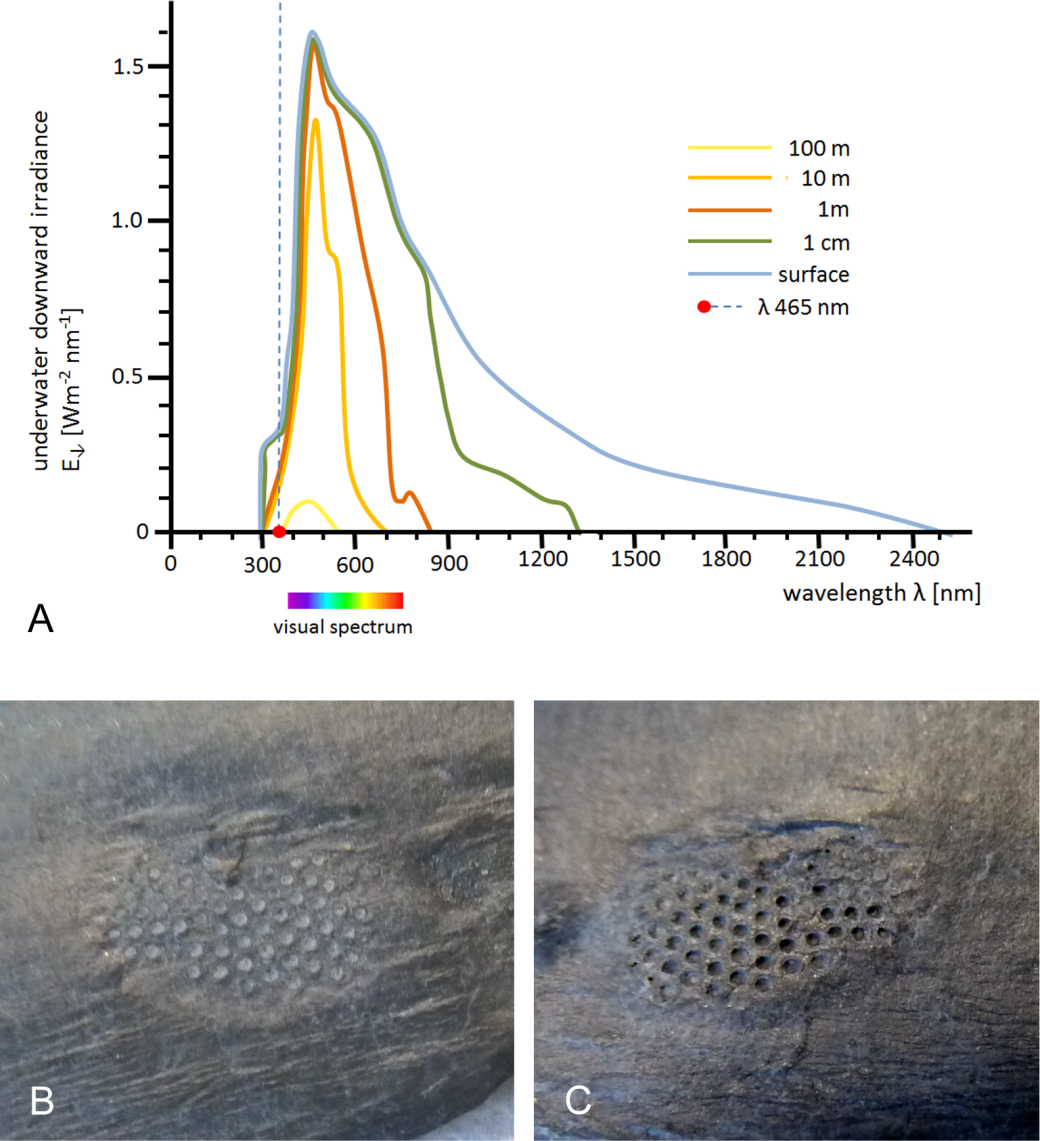
Underwater downward irradiance and fluorescence of trilobite lenses under UV-A light and day-light conditions. (A) Underwater downward irradiance (changed and simplified after Wozniak & Dera, 2007). (B) Isolated moult of a *Chotecops* compound eye with lenses preserved (GIK 2118) showing a very slight fluorescence in the calcitic lenses of the trilobite compound eye when illuminated with UVA-light (∼365 nm) under day-light conditions. (C) Isolated moult of a *Chotecops* compound eye with lenses preserved (GIK 2119) showing a very slight fluorescence in the calcitic lenses of the trilobite compound eye when illuminated with UVA-light (365 nm) under day-light conditions, but not as evident as in (B). (B, C) scale bar ∾ 1 mm.

It is well known that clear seawater has a transmission maximum at about 470 nm (Fig. 3A), so everything a trilobite living at a depth deeper than a few meters saw would appear in a blue-greenish light. UV-A light is attenuated roughly three times faster than blue light, making underwater environments contain much less UV than terrestrial habitats. Already above the water surface there is much less UV than blue—on a sunny day there is about four times as much blue (470 nm) as there is UV (365 nm).

The photoluminescence of the calcitic lenses in trilobites may have enhanced the width of the exploitable spectrum of vision of their bearers, transforming the UV-light into a fluorescence. We do not know the exact contents of impurities of the original calcitic lenses, thus nothing about the likely exact colour of a potential fluorescence. By physical reasons mentioned previously, it may be assumed that the early photoreceptors were sensitive to blue light, as are most photoreceptors of aquatic animals still today, which would match the blueish green fluorescence as shown in our experiment. If, at this early time in the evolution of complex marine animals, specialised UV-receptors had not yet originated by transforming UV-A by fluorescence of the lenses overlying the receptor system into blue-greenish light, it might have been possible to ‘catch’ these shorter wavelengths. This would extend the normal range of wavelengths available for vision, but without requiring specialised blue–green receptors. An argument against this facility is that there seems to be a general rule in (underwater) visual ecology that where UV-A is available in a given optical environment, there the animals have also UV-A sensitive photoreceptors, and only those animals do not have UV-A receptors which live in a UV-A deficient environment. So, why should trilobites would be an exception of this rule, and all the more, since during their 270-million-year history they could have been able to develop UV-A sensitive receptors, similarly to those of many recent marine animals. Furthermore, probably it would have ‘cost’ less to establish UV-A sensitive cells rather than a calcitic lens. But although we do have the calcitic lenses, we know nothing about the properties of the receptor cells below, and it is likely that any UV-A-induced fluorescence in this system would have produced images of very poor quality because of a drastically reduced contrast.

Regarding other visual systems of today, it is well known that in all known recent visual systems the amount of light scattered diffusely in the dioptric media (cornea, lens, crystalline cone, etc.) is minimized. In the human eye, for example, light is scattered diffusely in the vitreous body, which gives a non-imaging interior light field; this is greatly disadvantageous for image formation. One of the functions of the retinal pigment epithelium (containing melanin between the chorioid and retina) is to absorb this vitreous-scattered light.

The cuticular microstructure of the trilobites’ exoskeleton has been explored by several workers. It is generally agreed that the cuticle consists of the following layers (i) a very thin, originally organic layer, not often preserved, and sometimes phosphatised, (ii) a thin outer layer, often prismatic, with the crystallites arranged perpendicular to the surface. This outer layer, in *Asaphus* is about 1/15th of the total thickness of the cuticle (Dalingwater, 1973) (iii) a much thicker principal layer with distinct laminations, parallel with the outer and inner surfaces. This, like the outer layer, consists of low magnesian calcite (Wilmot & Fallick, 1989). Dalingwater & Miller (1977). Note that in the principal layer “Individual calcite crystals are difficult to resolve, but roughly shaped perpendicular plates of calcite (*...normal to the cuticle surface...*) are prominent... in some cases pierced by canal-like elements”. Likewise Dalingwater et al. (1991) comment that the principal layer “consists of fine crystallites, presumably of calcite, sometimes with their long axes arranged roughly perpendicular to the cuticle surface.” Wilmot (1990) notes that trilobite cuticles were able to resist both tensile and compressive forces. The outer, prismatic layer was able to resist compressive forces acting normal to the surface. The principal layer, on the other hand, with its small crystals, acted as a crack-stopper as well as providing bulk to the exoskeleton.

In other words, the principal layer consists of small calcite crystals, sometimes with a rough orientation perpendicular to the surface.

Calcite in trilobite eyes was likewise orientated so that its *c*-axis was parallel to the optical axis of the lens and perpendicular to the surface. This ordered calcite orientation minimized the optical problem caused by the birefringence of calcite. The eyes are only a specialised part of the exoskeleton, and the orientation of the *c*-axes in the lenses are concordant with the overall structure of the cuticle. However, the calcite crystals in the exoskeleton should also transfer UV-A to blue-green light. Thus, the whole body surface of trilobites illuminated by UV-A light should emit faint blue–green light, which could be very disadvantageous due to camouflage disruption: a trilobite emitting blue–green light would be visually very striking both for their prey and predators. Unfortunatly, the emission of this blue–green light in our fossils is so low that it cannot be photographed.

Thus, if calcite in lenses as found in the optical apparatus of trilobite compound eyes had such disadvantageous properties for any visual quality, and even the exoskeleton under UV-light may have been somewhat luminescent, evolution should somehow have eliminated these disruptive phenomena. A simple method to improve the quality of trilobite vision would have been to avoid the use of calcite in the dioptric apparatus altogether—recent arthropods use chitin instead.

Thus, there remain some interesting questions to be answered. Was fluorescence the reason why calcite has not been used more often in aquatic optical systems? And: why did the UV-A-induced photoluminescent blue–green glow in trilobite eyes and exoskeletons not cause problems for the trilobites?

A first strategy to escape from fluorescence would be to produce a calcite so pure that is does not contain any impurities. However, whether this was possible for a biological system remains doubtful.

Another effective strategy would be to avoid the UV-A light itself. Many trilobites probably have lived a crepuscular or nocturnal life (Clarkson, 1998), when their light environment was UV-A deficient. In particular, the early trilobites of the Cambrian and probably their predecessors were bottom dwellers. The invasion of the pelagic and planktonic realm by trilobites did not begin before the Furongian (upper Cambrian) and only was truly under way in the early Ordovician and later (McCormick & Fortey, 1998; Tortello & Esteban, 2003; Schoenemann et al., 2010; Tanaka et al., 2015), during the Great Ordovician Biodiversity Event.

Figure 3A shows the well-known optical fact that both UV-A/B/C and infrared light are strongly absorbed by (sea)water. As light propagates deeper and deeper into water, both the short (UV) and long (IR, red, green) wavelenghts are quickly absorbed, and depending on the water type, after a few decimeters/meters only quasi-monochromatic blue (∼475 nm) light remains. Due to this strong wavelength-selective absorption of water, the UV-A intensity of light is practically zero in water deeper than a few m or dm. The majority of trilobites surely lived deeper in the sea than a few m/dm.

Furthermore, many of the early trilobites presumably lived on organic material on or in the sea-floor sediment, and many of them preferred muddy ecosystems. When the mud was perturbed the water would become turbid. The optical haziness in sea water is caused by fine particles which scatter and absorb UV-A light very strongly. The intensity of the scattered light depends on the fourth power of the frequency, so blue and UV-light are scattered much more strongly than red light. As a consequence, the photoluminescence of their calcite lenses was visually irrelevant, because it was (more or less) not present in the environment of early trilobites.

Finally, one should bear in mind the conditions of radiance during the Palaeozoic, when the trilobites were living. It is well known that, due to the ozone layer being deficient or absent during the Archean, high energy radiation was able to penetrate more deeply into oceans than it does at present, and thus the potential damage rates to DNA were magnitudes higher than today. DNA damage must have been the principal factor for UV-induced mortality in the Archean oceans (Cockell, 1998; Cockell, 2000a; Cockell, 2000b; Cockell & Horneck, 2001). Thus at 5 m depth the potential DNA-damage rate may have been 2 orders of magnitude higher than today, and still one order higher at 15 m depth (Cockell, 2000a). A quite rapid change started probably ∼800 million years ago (Ma) (Qiu, 2014), and by at least 700 Ma oxygen levels might have been sufficient for respiration in metazoans (Margulis, Walker & Rambler, 1976; Bekker et al., 2004; Hessen, 2008). Having just about achieved an almost modern atmosphere ∼520 Ma, and probably due to the availability of certain minerals for the construction of shells of modern type (Cook & Shergold, 1984), the ‘Cambrian explosion’ became possible, and it was during this time that most modern clades originated (Margulis, Walker & Rambler, 1976; Cowen, 2005; Marshall, 2006; Hessen, 2008; Erwin et al., 2011). Trilobites appear in the Lower Cambrian among the oldest arthropod fossils, well equipped with a hard shell and complex compound eyes. As for many organisms of this era, the origin of trilobites probably lies before the ‘Cambrian explosion’ further back in the Proterozoic though without any fossil record, and we know little of the circumstances of radiation during the early evolution of the compound eyes of trilobites and their predecessors. Whether the invasion of the UV-A-deficient ecological niche as described was a consequence of the calcitic lenses, remains open, but is unlikely. It seems more realistic to assume that trilobites tracked regions rich in organic material easily to be digested, such as down in the muddy grounds of the ocean.

When the ozone levels rose during the late Proterozoic/early Palaeozoic the ozone levels rose, UV-B and UV-C were shielded almost completely, while UV-A was able to penetrate before this change, as it still does, and the amount of UV-A is comparable to that of today. But it surely is a good estimation to say that of the UV-A light just a small part causes fluorescence, while the rest passes through the thin lenses (∼200 µm) and UV-A light itself is just a small part of the light incident reaching the receptors. Furthermore, the percentage of non-UV-A light with respect to UV-A/B/C-light, both at present and at the beginning of the Cambrian was high enough that any ill-effects of fluorescence due to a low amount of UV-A were very minor relative to light of longer wavelengths transmitted through the lens. Figures 1C and 1E show the eyes and the lenses in ‘normal’ light, where fluorescence does not become apparent or does not occur. However, a slight blue fluorescence, inFig. 3B is evident where the same eyes are illuminated under day light, with UV-A light in the same way as under the same dark conditions in Fig. 1. One has to notice, of course, that under these conditions decades more of energy influenced the lenses. Fluorescence was not as evident in the second specimen under the same conditions, nor was it possible to cause any trilobite exoskeleton to glow. Whether the calcitic lenses originated even further back in time, when the UV-content was higher, cannot be confidently verified by any fossil record.

However, in this context it should be mentioned that the publication of Frank & Widder’s (1996) electrophysiological experiments on several species of deep-sea shrimp revealed unexpectedly high spectral sensitivity to UV light. Subsequent measurements of downward irradiance at 380 nm showed that UV of this wavelength was still detectable at 500–600 m, and this is indeed the depth at which these crustaceans live. Therefore, UV-relevant phenomenons seem to occur at deeper depths, but although the energy of the UV-light may be high enough to switch on highly sensitive receptor cells, it is possibly too low to evoke efficient fluorescent signals.

In summary, it is possible to answer the questions raised at the beginning.

The results show that there is a real potential for the lenses of trilobite eyes to show fluorescence (Fig. 1A). However, the optical and sensory consequences of fluorescence, as we have discussed, would, have been disastrous to the quality of vision because of a high loss of contrast (Fig. 2).

Fluorescence, however, is not the reason why calcite has not been used more often in aquatic optical systems. There are several reasons for this. The disadvantages of an optical system under UV-A light can be easily avoided by invading ecological niches which UV-light cannot influence—as indeed the early trilobites did. They were bottom dwellers, living on muddy sea floors where the water could readily become turbid when the substrate was stirred up, as would be expected for trilobites searching for organic material. Under such conditions, light of short wavelength was effectively scattered and absorbed. But the reason to invade this hazy part of the ocean in the first place, to where UV-A/B/C never passed through, was the availability of appropriate nutrients. This also answers the question, why the UV-A-induced photoluminescent blue–green glow in trilobite eyes and the camouflage-breaking properties of their exoskeletons did not cause problems for trilobites—it was not present in the environment that the early representatives preferred. So the calcitic lenses, with their great ability to focus light under water due to their high refractive index, probably originated in conditions where adverse stimulation caused by UV-light fluorescence was not a factor. Thus, the also important question—whether the fluorescent properties of calcitic lenses were a primary reason why calcite was never again used in underwater visual systems—can be answered very firmly with: no.

## Additional Information and Declarations

### Data Availability

The following information was supplied regarding data availability: The research in this article did not generate any raw data.
